# Identification of Candidate Anthocyanin-Related Genes by Transcriptomic Analysis of ‘Furongli’ Plum (*Prunus salicina* Lindl.) during Fruit Ripening Using RNA-Seq

**DOI:** 10.3389/fpls.2016.01338

**Published:** 2016-08-31

**Authors:** Zhi-Zhen Fang, Dan-Rong Zhou, Xin-Fu Ye, Cui-Cui Jiang, Shao-Lin Pan

**Affiliations:** Fruit Research Institute, Fujian Academy of Agricultural SciencesFuzhou, China

**Keywords:** transcriptome, *Prunus salicina* Lindl., anthocyanin biosynthesis, fruit ripening, biosynthetic enzyme, transcription factor

## Abstract

Anthocyanins are important pigments and are responsible for red coloration in plums. However, little is known about the molecular mechanisms underlying anthocyanin accumulation in plum fruits. In this study, the RNA-seq technique was used to analyze the transcriptomic changes during fruit ripening in the red-fleshed plum (*Prunus salicina* Lindl.) cultivar ‘Furongli’. Over 161 million high-quality reads were assembled into 52,093 unigenes and 49.4% of these were annotated using public databases. Of these, 25,681 unigenes had significant hits to the sequences in the NCBI Nr database, 17,203 unigenes showed significant similarity to known proteins in the Swiss-Prot database and 5816 and 8585 unigenes had significant similarity to existing sequences in the Kyoto Encyclopedia of Genes and Genomes and the Cluster of Orthologous Groups databases, respectively. A total of 3548 unigenes were differentially expressed during fruit ripening and 119 of these were annotated as involved in “biosynthesis of other secondary metabolites.” Biological pathway analysis and gene ontology term enrichment analysis revealed that 13 differentially expressed genes are involved in anthocyanin biosynthesis. Furthermore, transcription factors such as MYB and bHLH, which may control anthocyanin biosynthesis, were identified through coexpression analysis of transcription factors, and structural genes. Real-time qPCR analysis of candidate genes showed good correlation with the transcriptome data. These results contribute to our understanding of the molecular mechanisms underlying anthocyanin biosynthesis in plum flesh. The transcriptomic data generated in this study provide a basis for further studies of fruit ripening in plum.

## Introduction

The plum is one of the traditional fruit trees in China and is widely distributed in the world (Carrasco et al., 2012). Its fruit is highly appreciated by consumers. Plums are rich in bioactive substances such as vitamin C, carotenoids, polyphenols, and anthocyanins (Valero et al., 2013) and have great health benefits (Hooshmand and Arjmandi, 2009; Lee et al., 2009; Shukitt-Hale et al., 2009; Johnson et al., 2011). Color is one of the most important determinants of fruit quality. As a result of the influence of culture, Chinese people prefer red color. Anthocyanin accumulation is responsible for red coloration in plums (Cheng et al., 2015). Anthocyanins are widespread secondary metabolites that play an important role in the pigmentation of fruits. They are powerful antioxidants and naturally-occurring dietary anthocyanins are beneficial to human health (Nemie-Feyissa et al., 2015). Santhakumar et al. (2015) demonstrated that consumption of anthocyanin-rich plum juice attenuates thrombogenesis by reducing platelet activation/hyper-coagulability and oxidative stress. It is therefore, desirable to increase the anthocyanin content in plums, especially in the flesh, through improved cultivation methods, postharvest handling, or breeding.

The anthocyanin biosynthetic pathway has been well studied in many plants. Structural genes in the anthocyanin pathway, including those encoding phenylalanine ammonialyase (PAL), cinnamate-4-hydroxylase (C4H), 4-coumaroyl:CoA-ligase (4CL), chalcone synthase (CHS), chalcone isomerase (CHI), flavanone 3-hydroxylase (F3H), flavonoid 3′-hydroxylase (F3′H), flavonoid 3′,5′-hydroxylase, dihydroflavonol 4-reductase (DFR), anthocyanidin synthase/leucoanthocyanidin dioxygenase (ANS/LDOX), and UDP-glucose: flavonoid 3-O-glucosyltransferase (UFGT), have been isolated and characterized in plants (Tanaka et al., 2008). After the synthesizing in the cytosol, anthocyanins must be transported to the vacuole to exhibit their brilliant colors (Winkel-Shirley, 2001). This process is mediated by proteins including glutathione S-transferase (GST), multidrug and toxic compound extrusion, and ATP-binding cassette transporters (Hu et al., 2016).

Anthocyanin biosynthesis is cooperatively regulated by transcriptional regulators including MYB proteins, basic helix-loop-helix (bHLH) proteins, and WD40 proteins (Lepiniec et al., 2006; Feller et al., 2011). These regulators form an MBW complex that binds to promoters and activates transcription of structural genes of the anthocyanin biosynthetic pathway (Rahim et al., 2014; Li et al., 2016). The role of MBW in anthocyanin biosynthesis has been elucidated in fruit trees such as grape (Kobayashi et al., 2002, 2004; Walker et al., 2007), Chinese bayberry (Niu et al., 2010; Liu et al., 2013a,b), mangosteen (Palapol et al., 2009), blood orange (Crifò et al., 2011; Butelli et al., 2012), kiwifruit (Fraser et al., 2013), litchi (Lai et al., 2014, 2016), sweet cherry (Shen et al., 2014), apple (Takos et al., 2006; Espley et al., 2007; Xie et al., 2012; Chagné et al., 2013; An et al., 2015), and peach (Rahim et al., 2014; Zhou et al., 2014; Tuan et al., 2015).

In addition, other regulatory factors also affect anthocyanin biosynthesis via interaction with MBW complexex or by modulating the transcription of structural genes directly. MYBs that act as repressors of the anthocyanin pathway have been identified in several plants (Matsui et al., 2008; Salvatierra et al., 2013; Xu et al., 2013; Huang et al., 2014; Jun et al., 2015; Yoshida et al., 2015). Shin et al. (2007) found that PIF3 and HY5 regulated anthocyanin synthesis by binding directly to promoters of anthocyanin biosynthetic genes in *Arabidopsis thaliana*. The *Arabidopsis* COP1/SPA complex represses anthocyanin accumulation by degrading PAP1 and PAP2 proteins (Maier et al., 2013; Maier and Hoecker, 2014). Li et al. (2012b) also demonstrated that MdCOP1 negatively regulates accumulation of anthocyanin in apple peel by modulating the degradation of the MdMYB1 protein. ANAC078 has been shown to promote accumulation of anthocyanins by inducing the expression of flavonoid biosynthesis genes under high-light (Morishita et al., 2009). Recently, Zhou et al. (2015) found that NAC transcription factor BL, which controls the red-fleshed trait, interacts with PpNAC1 to form a heterodimer, and activate the transcription of *PpMYB10.1* to induce anthocyanin accumulation. This process is repressed by a SQUAMOSA promoter-binding protein-like (SPL) transcription factor PpSPL1. SPLs have been shown to inhibit the expression of anthocyanin biosynthetic genes and negatively regulate anthocyanin accumulation through destabilization of MBW (Gou et al., 2011). MADS-box genes are reported to be involved in regulation of anthocyanin accumulation (Jaakola et al., 2010; Wu et al., 2013). Jasmonates induce anthocyanin biosynthesis through degradation of jasmonate-ZIM-domain (JAZ) proteins and the subsequent release of MBW (Qi et al., 2011). DELLA proteins promote anthocyanin biosynthesis by sequestering MYBL2 and JAZ proteins, which repress the activity of MBW, to release bHLH/MYB subunits in *Arabidopsis* (Xie et al., 2016). Furthermore, epigenetic mechanisms also play important roles in anthocyanin biosynthesis (Wang et al., 2013; Zabala and Vodkin, 2014).

The ‘Furongli’ plum (*Prunus salicina* Lindl.), a red-skinned and red-fleshed cultivar, is native to Fujian, where it has been cultivated for more than 700 years. Fruit of ‘Furongli’ is popular for its attractive color, delicious taste, and health-promoting nutrients (Fang et al., 2014). The fruit can be eaten fresh and is used for the production of candied fruits. In recent years, RNA-seq-based transcriptome analysis has been extensively used for identification of functional genes in fruit trees. Rodamilans et al. (2014) analyzed the transcriptome changes in response to *Plum pox virus* infection in *P. cerasifera*. Jo et al. (2015) reported the leaf transcriptome assembly of two different *P. salicina* cultivars. More recently, González et al. (2016a) reported the fruit skin transcriptomes of two Japanese plum cultivars with different skin color and developed candidate EST–SSR markers for marker-assisted selection of fruit skin color in the Japanese plum (González et al., 2016b). In the present study, we analyzed the transcriptomic changes during the ripening of ‘Furongli’ plum fruits to identify candidate genes involved in the biosynthesis of anthocyanins. Based on the RNA-seq datasets generated, we identify several potential structural genes, and transcription factor genes related to anthocyanin biosynthesis. The transcriptomic data generated in this study provide a basis for further studies of fruit ripening in plum, and identify candidate genes involved in sugar accumulation, organic acid degradation, fruit softening, and pigmentation.

## Materials and methods

### Plant materials

**Figure 1:**
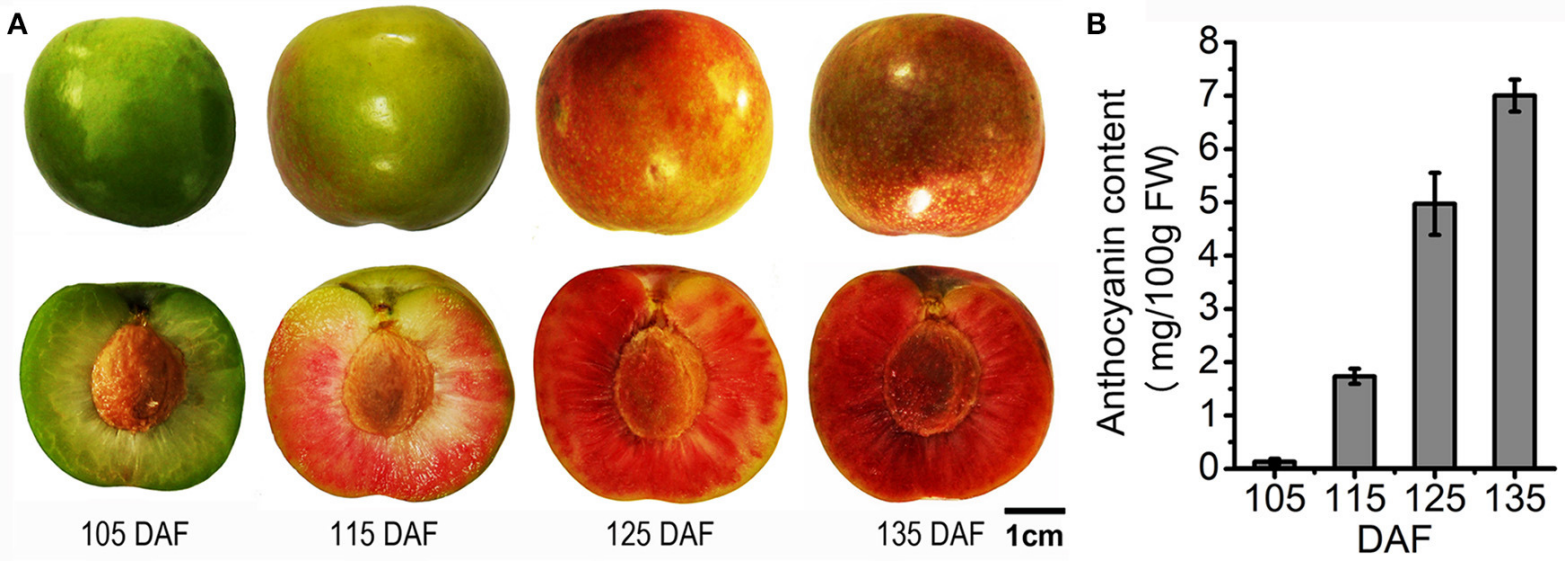
**Images of ‘Furongli’ plum and anthocyanin content in the fruits. (A)** ‘Furongli’ plums at different time-points selected for sequencing. Representative photographs of plums at 105, 115, 125, and 135 days after flowering (DAF). **(B)** Anthocyanin content in plums at different ripening stages. The vertical bars represent the standard error of triplicate experiments.

All samples were collected from 5-year-old field grown ‘Furongli’ plum (*Prunus salicina* Lindl.) trees in an orchard in Yongtai County, Fujian Province, China. Fruit samples were harvested at 105, 115, 125, and 135 days after flowering (DAF) from three trees in 2014 (Figure 1A). Twenty representative fruits were sampled from each tree at each developmental stage and sliced. The sliced samples were combined and immediately frozen in liquid nitrogen and kept at −80°C until use.

### Determination of anthocyanin content

Anthocyanin content was quantified as described by Niu et al. (2010). Briefly, approximately 3 g of sample was ground to a fine powder in liquid nitrogen and extracted with 20 mL extraction solution (0.05% HCl in methanol) at 4°C for 24 h. After centrifugation at 8000 × g for 20 min, the supernatant was transferred into a clean tube. One milliliter of supernatant and 4 mL of either bufferA (0.4MKCl, adjusted to pH 1.0 with HCl) or buffer B (1.2 N citric acid, adjusted to pH 4.5 with Na_2_HPO_4_) were mixed and the absorbance at 510 and 700 nm (*A*_510_ and *A*_700_) measured for A and B buffers, respectively. The anthocyanin content was calculated according to Romero et al. (2008) using the following formula: TA = *A* × MW × 5 × 100 × *V*/*e*, where TA stands for total anthocyanin content (mg/100 g, as cyanidin-3-O-glucose equivalent), *V* for final volume (mL), and *A* = [*A*_510_ (pH 1.0) − *A*_700_ (pH 1.0)] − [*A*_510_ (pH 4.5) − *A*_700_ (pH 4.5)]. A molar absorptivity (*e*) of 26,900 and molecular weight (MW) of 449.2 were used according to Wrolstad et al. (1982). Three measurements were taken for each biological replicate.

### RNA preparation, library construction, and RNA-seq

Total RNA was extracted from each sample using a EZNA Plant RNA Kit (Omega Bio-tek). The concentration of RNA was quantified with a Qubit 2.0 Fluorometer (Invitrogen, Life Technologies, CA, USA), and RNA integrity was evaluated using an Agilent 2100 Bioanalyzer (Agilent Technologies, Santa Clara, CA, USA). Equal amounts of RNA from three samples at the same stage were mixed together. The RNA isolation, library construction, and RNA-seq were performed by staff at Beijing BioMarker Technologies (Beijing, China). cDNA libraries were constructed as described by Han et al. (2013). Poly-A mRNA was enriched using poly-T oligoattached magnetic beads, then broken into small pieces, and used as template for cDNA synthesis. First strand cDNA was synthesized using reverse transcriptase and random primers, followed by second strand cDNA synthesis using DNA Polymerase I and RNase H. The cDNA libraries were sequenced on an Illumina HiSeq™ 2500.

### *De novo* transcriptome assembly

Before assembly, the raw reads in FASTQ format were filtered using in-house Perl scripts to discarding the reads containing the sequencing adapters and low-quality reads with ambiguous “N” bases or in which more than 10% of bases had a *Q* ≤ 20. The left files (read1 files) from all libraries/samples were pooled into one large left.fq file, and right files (read2 files) into one large right.fq file. *De novo* assembly of the clean reads was performed using Trinity (Grabherr et al., 2011) with min_kmer_cov set to 2 by default and all other parameters set to their default values.

### Functional annotation and classification

To annotate unigene sequences of ‘Furongli’ plums, blastx search (*E* < 10^−5^) was used to search against the NCBI nonredundant protein (nr), UniProt/Swiss-Prot, Gene Ontology (GO), Cluster of Orthologous Groups of proteins (COG), and Kyoto Encyclopedia of Genes and Genomes (KEGG) and Swiss-Prot databases and retrieve protein functional annotations based on sequence similarity. GO terms were assigned to the unigenes using Blast2GO (Conesa et al., 2005) with *E* ≤ 10^−5^. The distribution of the GO functional classifications of the unigenes was plotted using WEGO software (Ye et al., 2006).

### Unigene differential expression analysis

Reads from the four cDNA libraries were mapped to the assembled unigenes using Bowtie (Langmead et al., 2009). Unigene expression levels were quantified using fragments per kilobase of transcript per million mapped reads (FPKM). FPKM values were calculated using RSEM (Li and Dewey, 2011). For each sequenced library, the read counts were adjusted using edgeR (Robinson et al., 2010) with one normalization factor. Differential expression analysis of two samples was performed using the DEGseq R package (Wang et al., 2010). Unigenes differentially expressed between two samples were screened using false discovery rate (FDR) < 0.01 and absolute log_2_ fold changes ≥1 as the threshold.

### Prediction of transcription factors

To identify transcription factors expressed during ripening of ‘Furongli’ plum fruit, predicted peptide sequences of all unigenes were searched against transcription factors in PlantTFDB 3.0 using the Transcription Factor Prediction module (http://planttfdb.cbi.pku.edu.cn/prediction.php) with default parameters.

### Correlation analysis of structural genes and transcription factors

Correlation analysis of anthocyanin structural genes and transcription factors was performed as described by Ye et al. (2015). To exclude false positives, we only selected unigenes with a FPKM value ≥10 in at least one of the four stages during fruit ripening. Transcription factors with correlation coefficient values of ≥0.95 by *t*-test were considered to have an expression that was significantly correlated with the expression of genes in anthocyanin biosynthetic pathways. The formula used was *t* = (*r*√(*n*–2))/√(1–*r*^2^), at *P* < 0.05 and *n* = 4. A value of |*t*|>*t*_0.05, 2_ = 4.303, implying *r* > 0.95, means significant correlation. Coexpression analysis was carried out using the “CORREL” function in Microsoft Excel 2003 and confirmed using in-house Perl scripts and IBM SPSS statistics software.

### Real-time quantitative RT-PCR analysis

Nineteen candidate differentially expressed genes involved in anthocyanin biosynthesis were selected for validation by real-time quantitative RT-PCR (qRT-PCR). Total RNA from fruit samples was extracted using a modified CTAB method as described by Xuan et al. (2015). The primer sequences used for qRT-PCR are listed in Table S1. The cDNA was transcribed from 500 ng of total RNA using the PrimeScript RT reagent kit with gDNA Eraser (Takara, Dalian, China). Quantitative RT-PCR was performed using the Eppendorf RealPlex^4^ real-time PCR system (Hamburg, Germany) in a total volume of 20 μL in each well containing 10 μL of 2 × SYBR Premix Ex Taq™ II (Tli RNaseH Plus, TaKaRa), 1 μL of cDNA (in 1:10 dilution), and 0.4 μL 10 μM primers. Quantitative PCR conditions were 5 min at 95°C, followed by 40 cycles of 5 s at 95°C, 15 s at annealing temperatures listed in Table S1, and 30 s at 72°C, followed by 60–95°C melting curve detection. The actin gene was used as the reference. The expression levels were calculated as described by Fang et al. (2013). Three biological and three technical replicates were performed.

## Results

### Anthocyanin accumulation during ripening of ‘Furongli’ plum fruits

As indicated in Figure 1A, the color of ‘Furongli’ plums changed from green to red during ripening and the flesh became pigmented before the skin. Anthocyanin accumulation is responsible for the red color of plums and the major anthocyanins in ‘Furongli’ plums are cyanidin 3-rutinoside and cyanidin 3-glucoside (Usenik et al., 2009; Zhang, 2009). The anthocyanin content of ‘Furongli’ plums increased from 0.13 mg/100 g FW to 7.0 mg/100 g FW as ripening proceeded (Figure 1B).

### RNA-seq and *de novo* transcriptome assembly

Four cDNA libraries were constructed from the total RNA of ‘Furongli’ plums at 105, 115, 125, and 135 DAF. These libraries were subjected to RNA-seq using an Illumina HiSeq2500, generating 41,555,484, 43,021,234, 40,991,934, and 37,023,409 raw 100-bp paired-end raw reads, respectively (Table 1). All of the raw reads are available in the NCBI SRA database (accession number SRP076001). After removing low-quality reads and trimming adapter sequences, 41,067,827, 42,872,666, 40,886,898 and 36,927,707 clean reads were obtained for the 105, 115, 125, and 135 DAF libraries, respectively.

**Table 1:** **Summary of sequencing and *de novo* assembly**.

**Sequences**	**105d**	**115d**	**125d**	**135d**
**BEFORE TRIMMING**				
Total nucleotides (bp)	8,394,207,768	8,690,289,268	8,280,370,668	7,478,728,618
Number of raw reads	41,555,484	43,021,234	40,991,934	37,023,409
**AFTER TRIMMING**				
Number of clean reads	41,067,827	42,872,666	40,886,898	36,927,707
GC content (%)	46.96	47.07	47.03	46.54
Q30 percentage (%)	86.11	86.02	86.46	87.49
**AFTER ASSEMBLY**				
Number of transcripts of combined data	100,711			
Number of unigenes of combined data	52,093			
Total nucleotides (nt) of transcripts (bp)	132,974,754			
Total nucleotides (nt) of unigenes (bp)	45,459,242			
Mean length of transcripts (bp)	1320			
Mean length of unigenes (bp)	872			
N50 of unigenes (bp)	1675			

**Figure 2:**
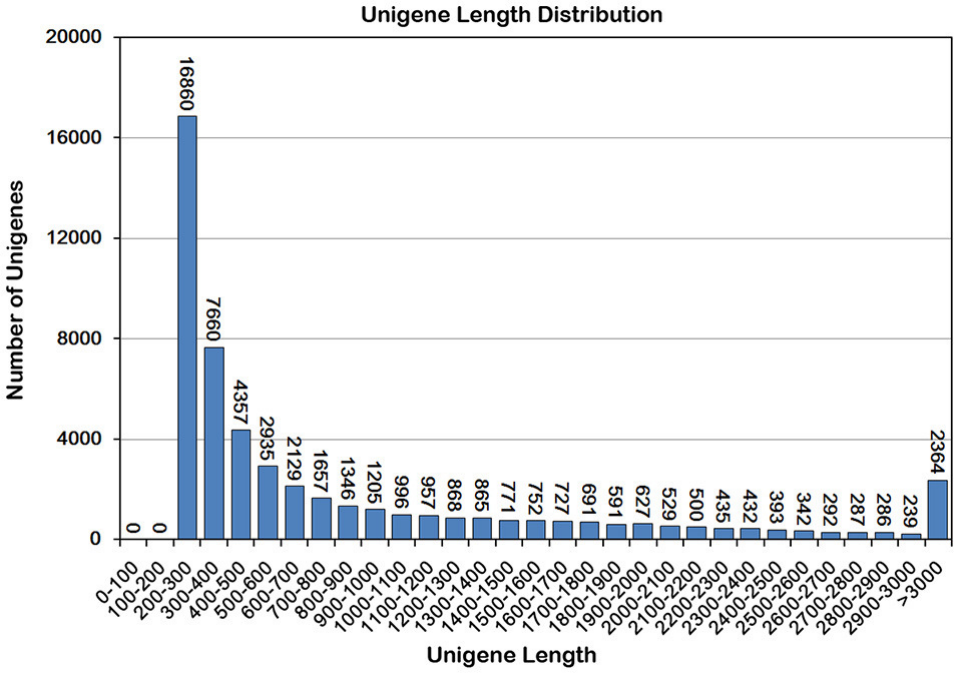
**Sequence length distribution of the unigenes in ‘Furongli’ plum fruit transcriptomes**. The *x*-axis indicates unigene length interval from 200 bp to > 3000 bp. The *y*-axis indicates the number of unigenes of each given sequence length.

Using Trinity, the clean reads from the four libraries were assembled into 100,711 transcripts with an average length of 1320 bp, and 52,093 unigenes with an average length of 872 bp were obtained (Table 1). Unigenes shorter than 500 bp accounted for 55.43% of the total unigenes and 26.77% of the unigenes (13,944) were longer than 1 kb (Figure 2). These results suggest that the quality of the unigene data was high enough for the following analyses.

### Functional annotation and classification

**Figure 3:**
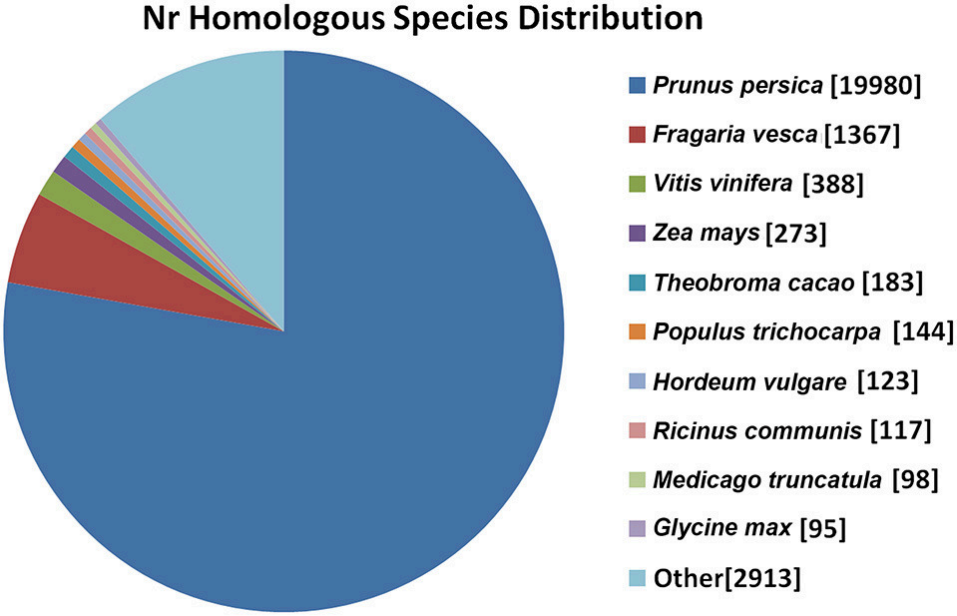
**Species distribution of the BLAST search results in nr database**. This figure shows the species distribution of unigene BLAST results against the nr database with a cutoff *E*-value of 10^−5^. Different species are indicated by different colors.

To annotate the transcriptome of ‘Furongli’ plums, 52,093 unigenes were searched against five public databases (nr, UniProt/Swiss-Prot, GO, COG, and KEGG) with a cutoff *E*-value of 10^−5^. The functional annotation results are listed in Table 2. Only 49.4% of the unigenes (25,730) were identified. The remaining unigenes (50.6%) could not be annotated with known genes (Table 2), most likely because oflimitations in the genomic information and the presence of short sequences. Only 947 (3.6%) of the unannotated unigenes were longer than 1000 bp, while 47.1% were shorter than 300 bp. The species distribution of the best match results in nr is indicated in Figure 3. The ‘Furongli’ plum unigenes showed the closest matches with *Prunus persica* (77.80%), followed by *Fragaria vesca* (5.32), *Vitis vinifera* (1.51), *Zea mays* (1.06), *Theobroma cacao* (0.71), *Populus trichocarpa* (0.56), *Hordeum vulgare* (0.48), *Ricinus communis* (0.46), *Medicago truncatula* (0.38), and *Glycine max* (0.37%).

**Table 2:** **Summary of functional annotations for the assembled ‘Furongli’ plum unigenes**.

**Database**	**Number of annotated unigenes**	**Percentage of annotated unigenes (%)**	**Length ≥ 300 bp**	**Length ≥ 1000 bp**
Nr	25,681	49.3	21,253	12,996
Swiss-Prot	17,203	33.0	14,686	9628
GO	18,623	35.7	15,895	10,639
COG	8585	16.5	5534	5534
KEGG	5816	11.2	4833	3141
All	25,730	49.4	21,277	12,997

**Figure 4:**
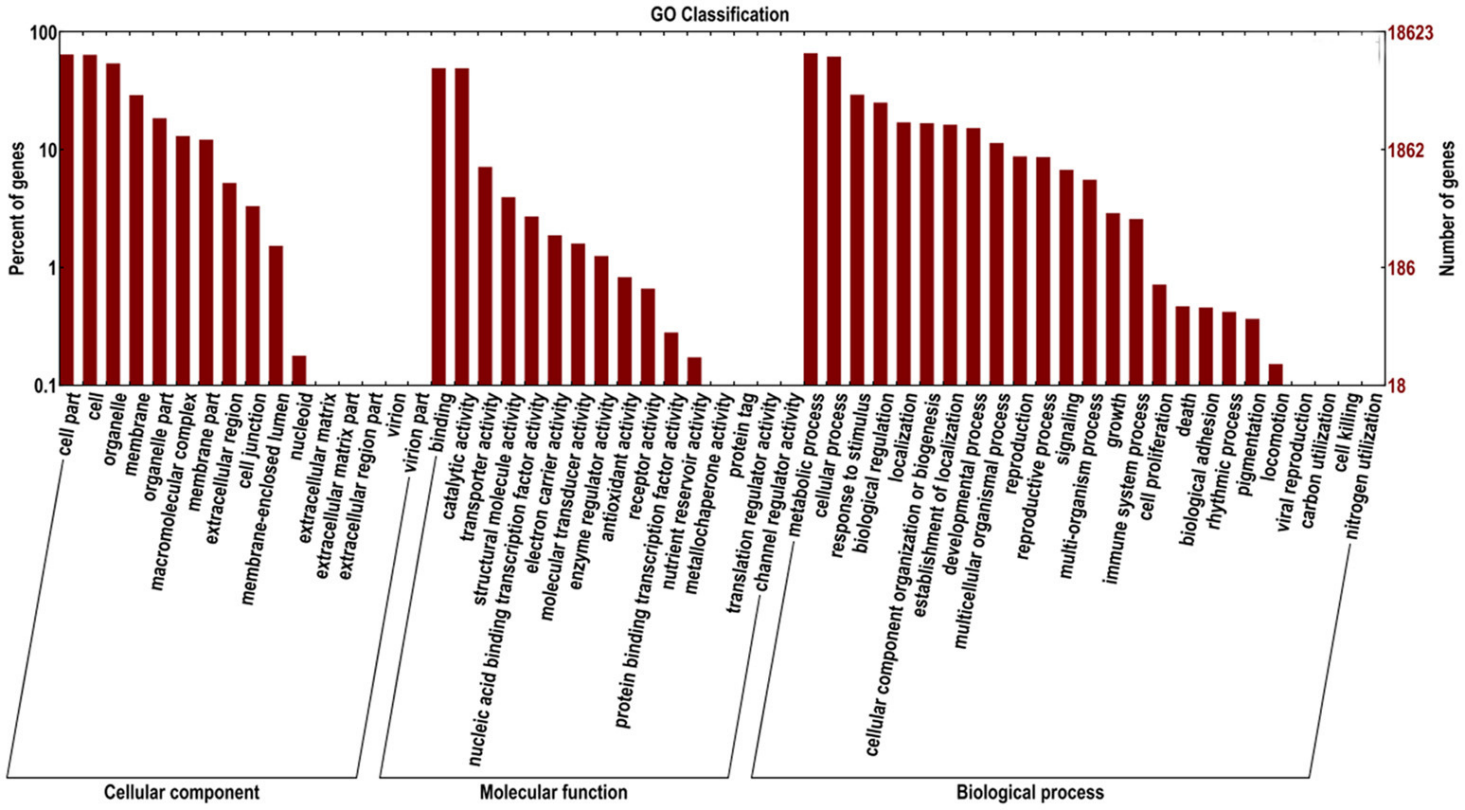
**GO classifications of ‘Furongli’ plum unigenes**.

The ‘Furongli’ plum unigenes were searched against the GO database to classify standardized gene functions. At least one GO term was assigned to 18,623 of the unigenes (Table 2). Unigenes were assigned to three main GO categories, including biological process category, cellular component category, and molecular function category, and 58 subcategories shown in Figure 4. The terms “cell,” “cell part,” and “organelle” were dominant in the cellular component category, the term “binding” and “catalytic activity” was dominant in the molecular function category, and the terms “metabolic process” and “cellular process” were dominant in the biological process category.

**Figure 5:**
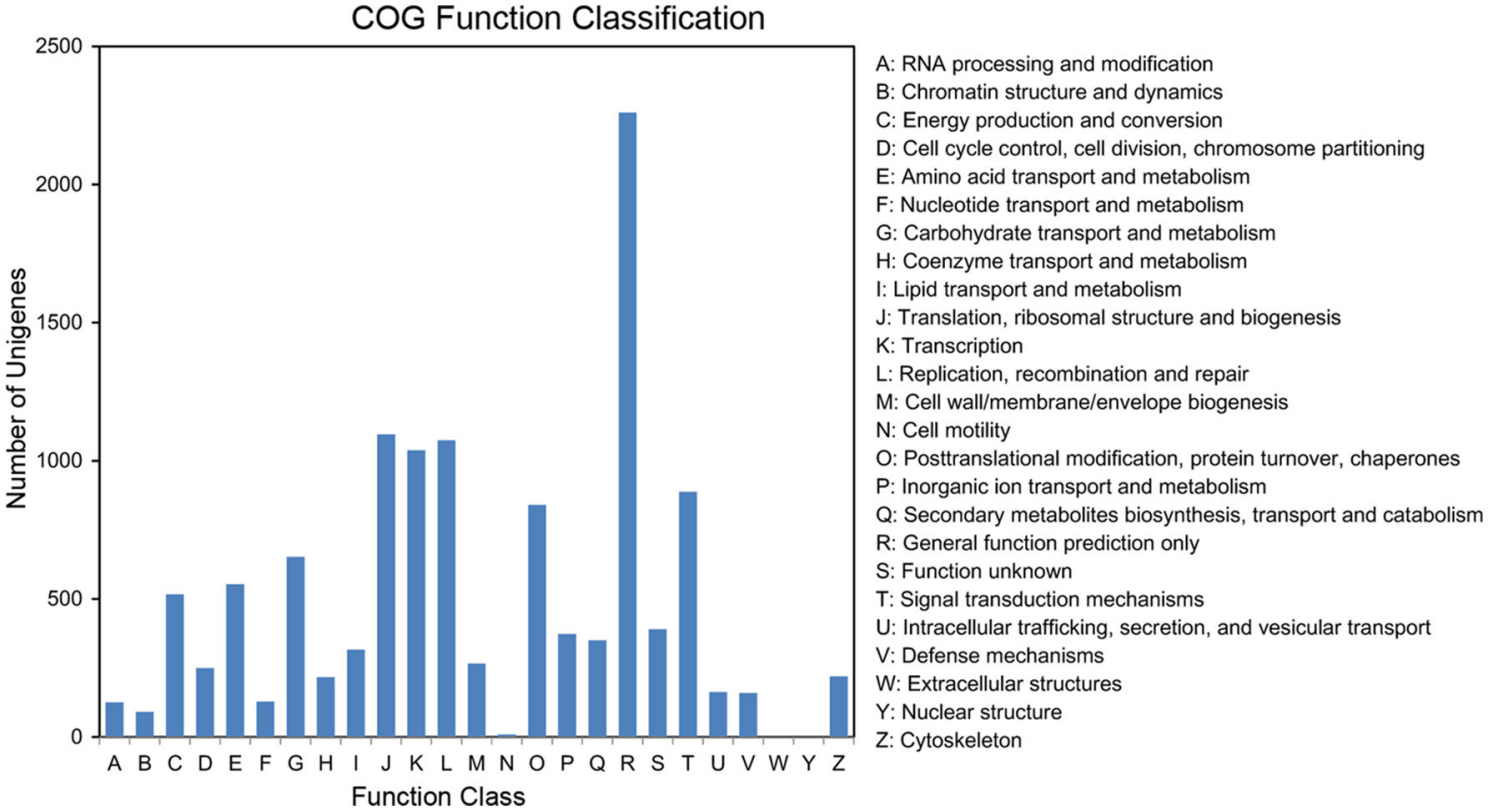
**Histogram of the COG classifications**. 8585 unigenes were grouped into 24 COG categories.

The completeness of the plum transcriptome library and the validity of the annotations were further evaluated by COG annotation. Out of the 25,730 annotated unigenes, 8585 (33.37%) were clustered into 24 COG categories (Figure 5). The cluster for “general functional prediction only” (2260, 26.32%) represented the largest group, followed by “translation, ribosomal structure and biogenesis” (1095, 12.75%), “replication, recombination, and repair” (1074, 12.51%), “transcription” (1038, 12.09%), “Signal transduction mechanisms” (887, 10.33%), “posttranslational modification, protein turnover, chaperones” (840, 9.78%), “carbohydrate transport and metabolism” (652, 7.59%), “amino acid transport and metabolism” (553, 6.44%), and “energy production and metabolism” (516, 6.01%). “cell motility” (9, 0.1%) and “nuclear structure” (1, 0.01%) represented the smallest groups. No unigene was assigned to “extracellular structures.”

**Figure 6:**
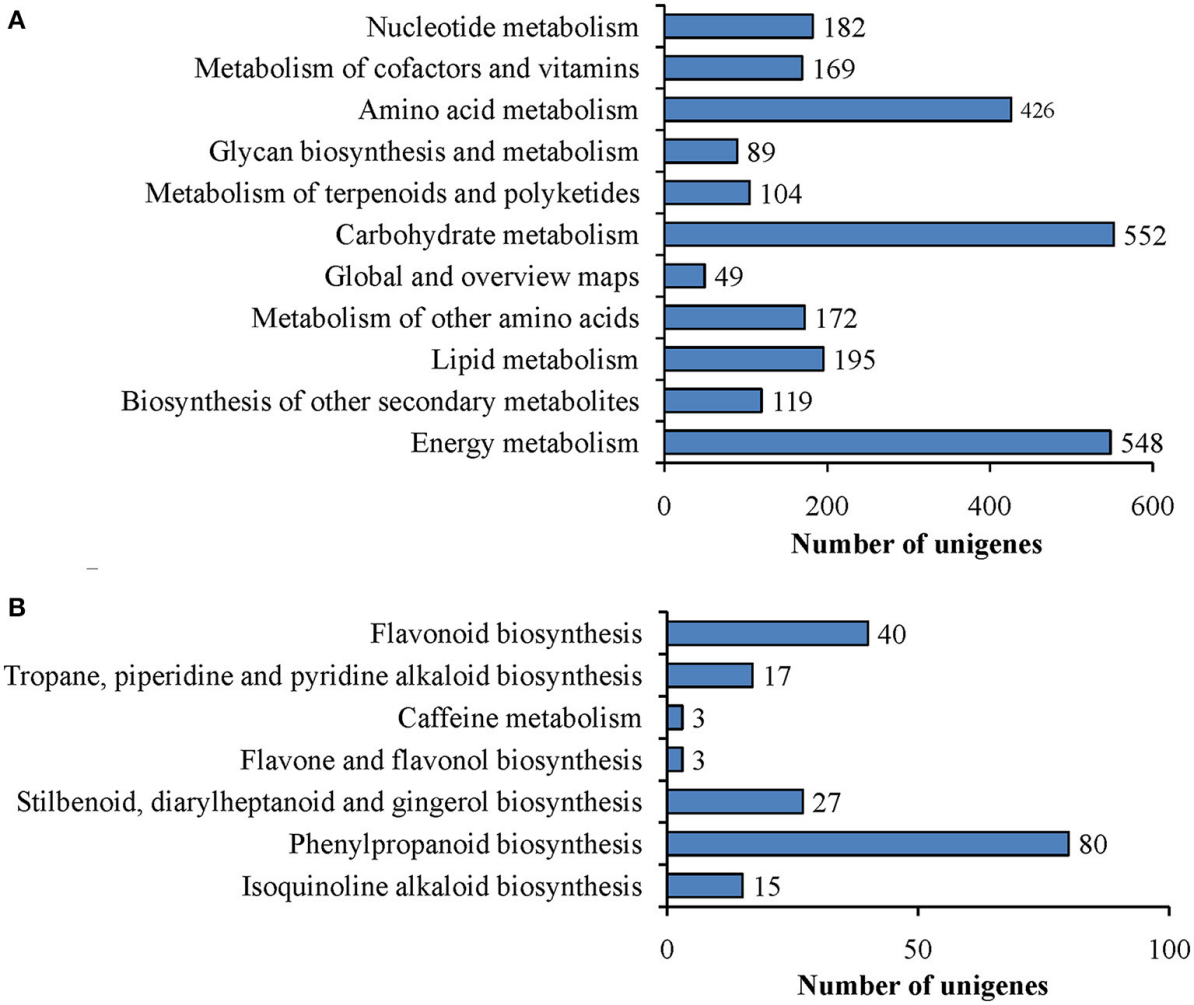
**Pathway assignment based on KEGG. (A)** Classification based on metabolism categories; **(B)** Classification based on “biosynthesis of other secondary metabolites” categories.

To better understand biological pathways involved in ripening of ‘Furongli’ plum fruits, all unigenes were searched against the reference canonical pathways in the KEGG database. Only 5816 (11.2%) unigenes had significant matches in the KEGG database and these were assigned to 115 KEGG pathways (Table S2). Among them, 2009 unigenes were assigned to metabolic pathways. As demonstrated in Figure 6A, 552 unigenes were assigned to carbohydrate metabolism, followed by Energy metabolism (548 unigenes), amino acid metabolism (426 unigenes), and lipid metabolism (195 unigenes). We concentrated on the “biosynthesis of other secondary metabolites” category in relation to fruit pigmentation. In this category, 119 unigenes were classified into seven subcategories (Figure 6B). Among these, the cluster for “phenylpropanoid biosynthesis” was the most represented, followed by “flavonoid biosynthesis.” “Flavone and flavonol biosynthesis” and “caffeine metabolism” appeared to be the smallest groups.

### Changes in gene expression during fruit ripening

**Figure 7:**
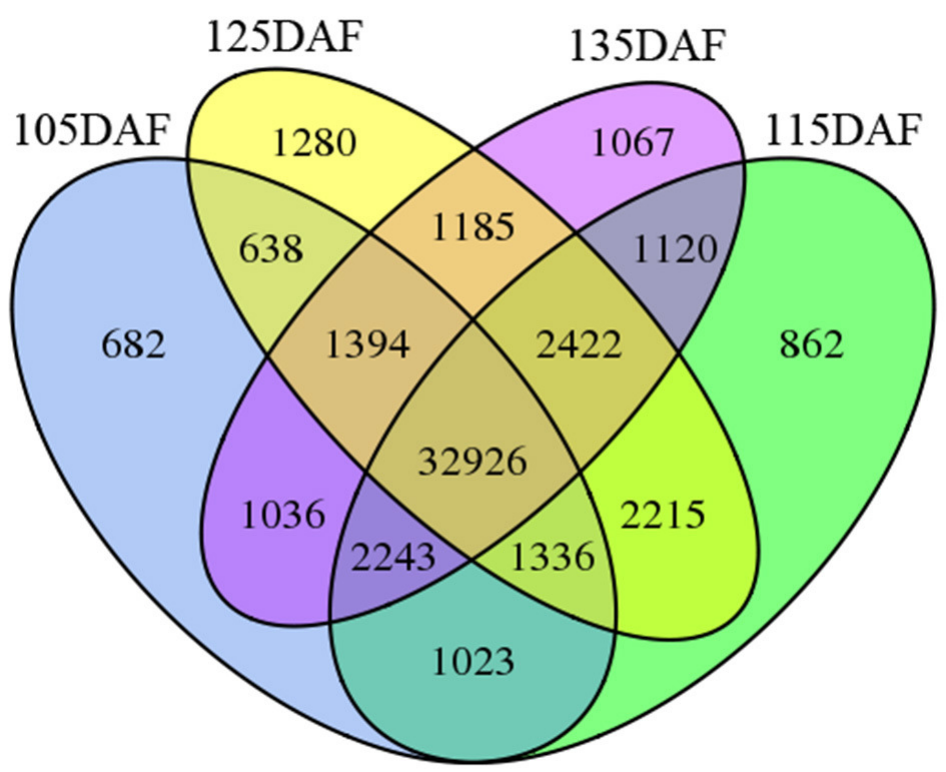
**Venn diagram illustrating the number of unigenes expressed at different developmental stages**.

To study unigene expression during different developmental stages, the reads from the four libraries were mapped to the assembled transcriptome. As indicated in Figure 7, most of the unigenes (32,926, 63.21%) were expressed in all four stages of development. A total of 3891 unigenes (7.47%) were only detected at a single stage of development. Of these stage-specific expressed unigenes, 682 (1.31), 862 (1.65), 1280 (2.46), and 1.067 (2.05%) unigenes were expressed exclusively at 105, 115, 125, and 135 DAF, respectively.

**Figure 8:**
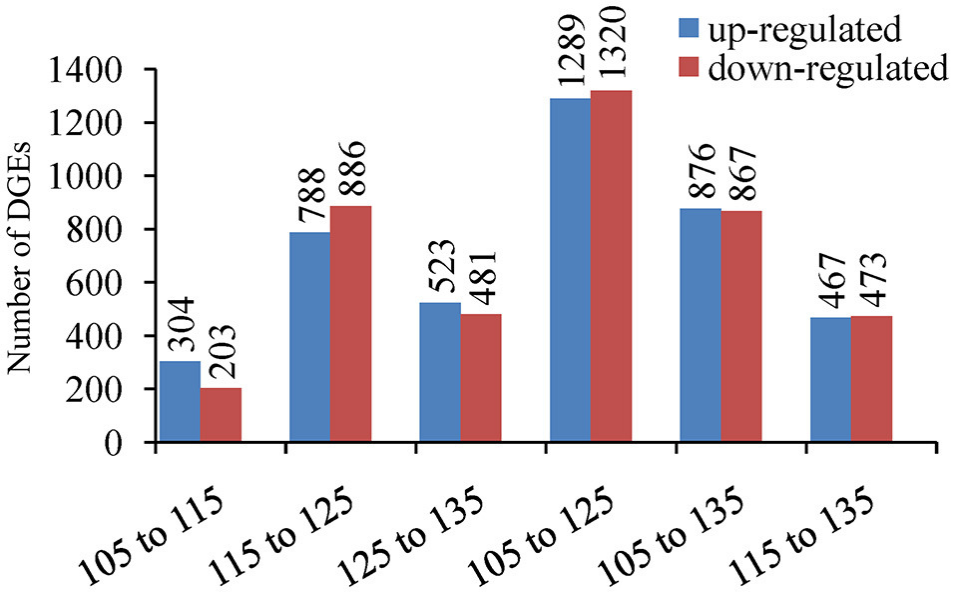
**Number of differentially expressed genes (DEGs) between 105, 115, 125, and 135 DAF in ‘Furongli’ plums**.

By comparing the libraries, 507 differentially expressed unigenes (304 upregulated and 203 downregulated) were identified between 105 and 115 DAF, with 1674 differentially expressed unigenes (778 upregulated and 886 downregulated) between 115 and 125 DAF, as well as 1004 differentially expressed unigenes (523 up-regulated and 481 downregulated) between 125 and 135 DAF (Figure 8). Between 105 and 125 DAF, there are 2609 differentially expressed unigenes (1289 up-regulated and 1320 downregulated) showed differential expression. Between 105 and 135 DAF, there were 1743 differentially expressed unigenes (876 upregulated and 867 downregulated) show different expression. Between 115 and 135 DAF, there are 940 differentially expressed unigenes (467 upregulated and 473 downregulated). When considered together, 3548 unigenes were differentially expressed during fruit ripening. This result suggests that the developmental period with the most dynamic changes in the transcriptome was between 115 and 125 DAF, with almost half of the differentially expressed genes (47.18%) showing significant changes during this period.

### Detection of candidate genes related to anthocyanin biosynthesis

To identify unigenes involved in anthocyanin biosynthesis, KEGG functional enrichment was analyzed to characterize the functions of differentially expressed unigenes (Table S3). A total of 11 unigenes encoding enzymes, including *PAL* (c38398.graph_c0), *C4H* (c23939.graph_c0), *4CL* (c30378.graph_c0), *CHS* (c37054.graph_c0), *CHI* (c28749.graph_c0), *F3H* (c23888.graph_c0), *F3*′*H* (c38186.graph_c0), *DFR* (c24831.graph_c0), *ANS*/*LDOX* (c29583.graph_c0), and *UFGT* (c19095.graph_c0), were assigned to the anthocyanin biosynthetic pathway based on KEGG (Table 3; Table S3). GO annotation was carried out to further identify anthocyanin-associated unigenes without annotation information in KEGG database (Table S4). The unigene c8193.graph_c0 (*CHI*) was assigned to “anthocyanin-containing compound biosynthetic process.” The unigene c25083.graph_c0 (flavonoid 3′-monooxygenase) was predicted to have flavonoid 3′, 5′-hydroxylase activity (GO:0033772). Furthermore, a *GST* (c29416.graph_c0), with best match to *AtGSTF12* (AT5G17220), also showed differential expression. As shown in Table 3, the expression of these 13 unigenes was significantly upregulated during fruit maturation.

**Table 3:** **Expression profiles of anthocyanin biosynthesis genes in ‘Furongli’ plums**.

**Gene name**	**Unigene ID**	**Gene length**	**FPKM**			
			**105 DAF**	**115 DAF**	**125 DAF**	**135 DAF**
*PAL*	c38398.graph_c0	2664	10.51	43.23	174.53	124.88
*C4H*	c23939.graph_c0	2195	45.98	89.76	273.74	150.46
*4CL*	c30378.graph_c0	2101	22.54	28.64	50.62	32.44
*CHS*	c37054.graph_c0	1946	5.46	24.49	127.20	169.99
*CHI*	c28749.graph_c0	1168	63.12	94.83	178.79	146.56
	c8193.graph_c0	1096	11.25	11.80	23.91	26.09
*F3H*	c23888.graph_c0	1598	60.75	128.48	251.98	178.00
*F3′H*	c38186.graph_c0	2020	253.50	354.89	1362.39	622.70
	c25083.graph_c0	2408	23.81	25.96	58.53	41.06
*DFR*	c24831.graph_c0	1476	2.55	2.73	12.28	16.92
*LDOX*/*ANS*	c29583.graph_c0	1673	245.80	1024.29	2328.40	1950.02
*UFGT*	c19095.graph_c0	1619	23.47	137.59	346.17	284.91
*GST*	c29416.graph_c0	994	78.55	540.53	1296.44	1247.29
*MYB*	c39005.graph_c0	1496	6.84	8.54	26.56	24.54
*MYBD*	c28480.graph_c0	1698	21.01	31.93	36.48	45.67
*bHLH*	c36695.graph_c0	3025	5.92	6.24	10.19	8.65
	c33382.graph_c0	1980	14.90	9.98	2.04	3.19
*NAC*	c27539.graph_c0	1674	4.02	8.02	16.91	18.54
	c19209.graph_c0	763	16.10	25.23	36.99	36.52

Transcription factors play important roles in the regulation of anthocyanin biosynthesis. In total, 791 unigenes (Table S5) were predicted to encode transcription factors from 55 different families (Table S6) and 147 of them were differentially expressed (Table S7). To identify transcription factors that were coexpressed with the candidate enzymatic genes involved in anthocyanin biosynthesis, a transcription abundance correlation analysis was carried out between the differentially expressed transcription factors and structural genes from the anthocyanin biosynthetic pathway. This identified 37 transcription factors whose expression levels were highly correlated with those of the candidate structural genes (Table 4; Table S8). Of these, 22 showed a significant correlation with five or more structural genes from the anthocyanin biosynthetic pathway. The identified transcription factors included homologs of *Arabidopsis* transcription factors that are implicated in regulating anthocyanin biosynthesis, such as MYB, bHLH, and NAC (Table 3).

**Table 4:** **Correlation analysis of structural genes involved in anthocyanin metabolism and transcription factors**.

**Gene ID**	**FPKM max**	**FPKM min**	**Description for the best hit in *A. thaliana***	**Number of correlations**
c39005.graph_c0	26.56	6.84	AtMYB113	6
c29499.graph_c0	40.87	8.06	AtMYB73	7
c32850.graph_c0	26.41	8.02	AtMYB102	3
c7988.graph_c0	30.47	13.12	CRY2-interacting bHLH 3	6
c18575.graph_c1	15.67	4.22	AtbHLH135	1
c19089.graph_c1	135.85	28.98	Transcription factor bHLH36	4
c19862.graph_c0	42.35	7.56	AtbHLH35	7
c33382.graph_c0	14.90	2.04	AtbHLH14	6
c36134.graph_c1	37.33	16.77	AtbHLH130	2
c38825.graph_c0	44.87	14.34	Zinc finger protein ZAT17	2
c33970.graph_c0	38.21	16.16	Zinc finger protein JACKDAW	5
c19087.graph_c0	342.48	92.52	AtNAC2	3
c19209.graph_c0	36.99	16.10	NAC domain containing protein 83	4
c27539.graph_c0	18.54	4.02	NAC domain-containing protein 100	6
c37766.graph_c1	16.22	7.19	NAC014	5
c24992.graph_c1	98.05	45.08	Ethylene response factor 61	3
c8431.graph_c0	301.81	132.51	Ethylene responsive element binding factor 5	1
c18924.graph_c0	424.36	180.10	Ethylene-responsive transcription factor	3
c24113.graph_c0	20.46	5.10	Ethylene-responsive element binding factor 13	8
c28635.graph_c0	71.57	10.92	FYF up-regulating 321 factor 1	6
c28868.graph_c0	140.98	50.99	Ethylene responsive element binding factor 1	4
c39002.graph_c0	24.73	4.64	C-repeat-binding factor 4	8
c34188.graph_c0	33.98	7.08	Cytokinin response factor 4	6
c30911.graph_c0	17.26	7.99	WRKY DNA-binding protein 4	3
c23690.graph_c0	13.81	5.05	WRKY transcription factor 29	9
c5911.graph_c0	11.66	3.24	bZIP transcription factor family protein TGA7	7
c14559.graph_c0	48.74	8.51	basic leucine zipper 9	9
c18932.graph_c0	402.53	106.55	basic leucine-zipper 44	8
c38252.graph_c0	370.27	89.02	basic leucine-zipper 44	6
c38790.graph_c0	65.16	10.19	basic leucine-zipper 6	6
c33394.graph_c0	13.24	5.35	AtC3H49	3
c35600.graph_c0	31.29	13.06	AtRNJ	8
c19054.graph_c0	179.42	84.98	Homeobox-leucine zipper protein HAT5	5
c34316.graph_c0	26.17	6.18	Homeobox-leucine zipper protein ATHB-13	6
c38928.graph_c0	23.59	10.91	Auxin response factor 17	5
c33782.graph_c0	10.53	4.47	Zinc finger protein constans-like 2	3
c28312.graph_c0	87.28	32.39	AP2/ERF and B3 domain-containing transcription repressor TEM1	4

A total of 37 MYBs were differentially expressed during ripening of ‘Furongli’ plums and three of them (c39005.graph_c0, c29499.graph_c0 and c32850.graph_c0) were associated with the anthocyanin biosynthetic pathway (Table 4; Table S8). The unigene c39005.graph_c0 (homologous to *AtMYB113*) was upregulated, while c29499.graph_c0 (homologous to *AtMYB73*) and c32850.graph_c0 (homologous to *AtMYB102*) were downregulated. The expression level of the homolog of *AtMYBD* (c28480.graph_c0) also increased. However, it was correlated with none of the structural genes. Seven of the differentially expressed transcription factors annotated as bHLH showed a significant correlation with anthocyanin biosynthetic genes (Table 4; Table S8). Only two of the *bHLH* genes (c7988.graph_c0 and c18575.graph_c1) showed a positive correlation with the expression of structural genes, while most of them were negatively correlated with that of structural genes involved in the anthocyanin biosynthetic pathway. In addition, a plum bHLH (c36695.graph_c0, log2 fold change < 1.0), which is the best BLAST match to *Arabidopsis* AtbHLH42, was significantly correlated with seven anthocyanin biosynthetic structural genes (Table S8). WD40 encoding unigenes (c28377.graph_c0 and c10590.graph_c1), which are the homolog of *AtTTG1*, did not show differential expression during the ripening process. Apart from the MBW components, other differentially expressed transcription factors, such as NAC, were also found to be potentially related to the anthocyanin pathway. Four plum *NAC* genes (c19087.graph_c0, c19209.graph_c0, c27539.graph_c0, and c37766.graph_c1) were significantly correlated with structural genes (Table 4; Table S8). The unigenes c27539.graph_c0 (a homolog of *AtNAC100*) and c19209.graph_c0 were upregulated during plum fruit ripening. Peach homolog of AtNAC100 have been reported to be involved in the regulation of anthocyanin accumulation in fruit flesh. GO annotation results indicated that c19209.graph_c0 is involved in “biological process: positive regulation of flavonoid biosynthetic process” (GO:0009963).

**Figure 9:**
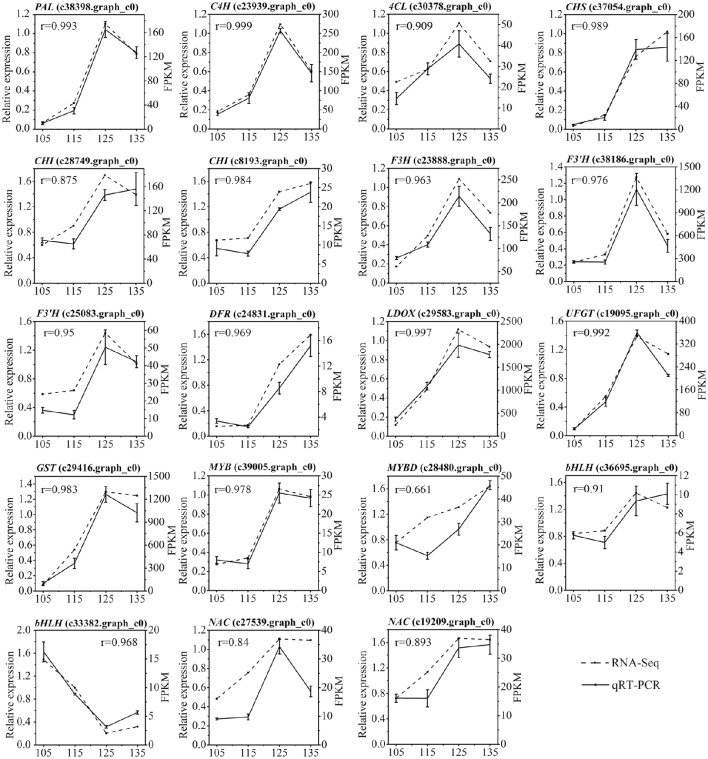
**Expression analysis of 19 differentially expressed genes related to anthocyanin biosynthesis in ‘Furongli’ plums during fruit ripening**. *Actin* was used as the internal control. The error bars represent the standard error of three biological replicates. The numbers above the graphics correspond to values obtained with the Pearson correlation. Pearson correlation between the RNA-seq data and qRT-PCR data was calculated using the log2 value of FPKM and the relative expression level.

We further analyzed the expression profiles of 19 candidate unigenes (13 structural genes and six transcription factors) involved in anthocyanin biosynthesis using qRT-PCR. The results indicated that there is a good correlation between RNA-seq data and qPCR data for most of the genes (Figure 9).

## Discussion

In this study, we constructed a transcriptome of ‘Furongli’ plums during fruit maturation. In total, 52,093 unigenes were assembled with a mean length of 872 bp, which is comparable to 944 bp for sweet cherry (*P. avium* L.) (Wei et al., 2015). A large quantity of genomic data is available for many rosaceous plants, but only 49.4% of the plum unigenes were annotated to public databases (nr, Swiss-Prot, GO, COG, and KEGG). This means that more than half of the unigenes have no significant known homologs. The low rate of annotated unigenes could be a result of limitations in the genomic information available for *P. salicina* Lindl. as is the case in other non-model plant species (Yates et al., 2014; Wei et al., 2015; Wu et al., 2015). As expected, 77.80% of the unigenes annotated using nr show significant similarity to *P. persica* transcripts. The unannotated unigenes could be plum specific genes with novel functions. The transcriptome of ‘Furongli’ plums will serve as an important datasets for studying plum ripening processes such as sugar accumulation, organic acid degradation, fruit softening, and pigmentation.

Anthocyanin-rich plums are of great interest for their implications in human health (Santhakumar et al., 2015). The main objective of this study was to identify genes involved in anthocyanin biosynthesis in plums. RNA-seq-based comparative transcriptome analysis has been shown to be an efficient strategy for the investigation of genes involved in anthocyanin biosynthesis in several plants, such as kiwifruit (Li et al., 2015a), sweet cherry (Wei et al., 2015), zoysiagrass (Ahn et al., 2015), anthurium (Li et al., 2015b), and potato (Liu et al., 2015). In the later ripening stages, ‘Furongli’ plums accumulate anthocyanins rapidly (Figure 1). The expression of anthocyanin biosynthetic genes has been shown to be correlated with fruit anthocyanin content in Rosaceae such as apple (Feng et al., 2013, 2014; Vimolmangkang et al., 2014), pear (Li et al., 2012a; Yang et al., 2015), sweet cherry (Wei et al., 2015), strawberry (Xu et al., 2014), and plum (Cheng et al., 2015). In the present study, changes in gene expression between different stages of ripening were analyzed to identify differentially expressed genes implicated in anthocyanin biosynthesis, including *PAL, C4H, 4CL, CHS, CHI, F3H, F3*′*H, DFR, ANS*/*LDOX, UFGT*, and *GST*, were significantly upregulated in the late stages of fruit maturation (Table 3; Figure 9).

Anthocyanin biosynthesis is regulated by several well-studied transcription factors such as MYB, bHLH, and WD40 (Gonzalez et al., 2008). MYB transcription factors have been reported to play a pivotal role in anthocyanin biosynthesis regulation in several fruit trees (Chagné et al., 2013; Ravaglia et al., 2013; Umemura et al., 2013; Lai et al., 2014; Shen et al., 2014; Tuan et al., 2015; Zhai et al., 2015; Jin et al., 2016). Lin-Wang et al. (2010) demonstrated that R2R3 MYBs are highly conserved in rosaceous plants and MYBs from European plum and cherry plum are able to induce the anthocyanin accumulation in tobacco. Gu et al. (2015) proposed that constitutive activation of *PcMYB10.6* is responsible for red pigmentation in purple-leaf plum. Cheng et al. (2015) indicated that *PsMYB10* was involved in ethylene-regulated anthocyanin biosynthesis in plums. These studies suggested that MYBs play a role in anthocyanin biosynthesis in the plum. In this study, a plum *MYB* (c39005.graph_c0) was positively correlated with structural genes. Its *Arabidopsis* homolog (AtMYB113) is an activator of the anthocyanin pathway. Recently, AtMYBD was shown to enhance anthocyanin biosynthesis by repressing the negative regulator *MYBL2* (Nguyen et al., 2015). The expression of an *AtMYBD* homolog (c28480.graph_c0) was upregulated in late ripening stages, but it does not show significant correlation with any structural genes. In addition, the anthocyanin biosynthetic pathway is also negatively controlled by MYB repressors in many plants, including Arabidopsis (Dubos et al., 2008; Matsui et al., 2008; Zhu et al., 2009), poplar (Yoshida et al., 2015), *Medicago truncatula* (Jun et al., 2015), grapevine (Cavallini et al., 2015; Pérez-Díaz et al., 2015), strawberry (Salvatierra et al., 2013), and apple (Lin-Wang et al., 2011). We found that two *MYB*s (c29499.graph_c0 and c32850.graph_c0) were negatively correlated with anthocyanin biosynthetic genes. Another important component of the MBW complex, bHLH proteins have been shown to be essential for or to enhance MYB-induced anthocyanin accumulation in transient expression assays (Liu et al., 2013b; Rahim et al., 2014; Feng et al., 2015; Starkevič et al., 2015; Wei et al., 2015; Lai et al., 2016). Our results indicated that c36695.graph_c0, which is highly homologous to AtTT8 (AT4G09820), accumulates to higher levels in late stages of ripening and shows significant correlation with anthocyanin biosynthetic genes. Conversely, the expression of c33382.graph_c0 was repressed as ripening proceeded. The unigene c33382.graph_c0 is a homolog of *AtbHLH14* (AT4G00870), which belongs to bHLH subgroup IIId. Song et al. (2013) demonstrated that *Arabidopsis* lines overexpressing *bHLH17* showed jasmonate-induced anthocyanin accumulation. NAC proteins have also been reported to be involved in anthocyanin synthesis in *Arabidopsis* (Morishita et al., 2009). Recently, a peach homolog of *AtNAC100* was shown to be responsible for regulation of anthocyanin accumulation in flesh (Zhou et al., 2015). Our results indicated that a plum homolog of *AtNAC100* (c27539.graph_c0) is upregulated and positively correlated with anthocyanin biosynthetic genes. It should be noted that coexpression analysis usually requires a large sample size and the small sample size in our study will reduce the reliability of our results. However, relatively small numbers of samples have recently been used to analyze the correlation of structural genes and regulators involved in biological processes, such as flavonoid biosynthesis (Zhai et al., 2015) and fruit ripening (Wu et al., 2016). The exact roles of these candidate transcription factor should be investigated in further studies.

In the current study, we used RNA-seq to analyze changes in the transcriptome during ripening of ‘Furongli’ plums. We generated 52,093 unigenes and over 50% of them were not annotated to public databases. Unigenes differentially expressed during fruit ripening were identified. Candidate genes encoding anthocyanin biosynthetic enzymes and transcription factors involved in anthocyanin biosynthesis were identified using functional annotation and coexpression analysis of differentially expressed genes. The expression patterns of some candidate genes encode anthocyanin biosynthetic enzymes and transcription factors were further validated by qRT-PCR. This provides an important datasets for studying fruit ripening processes, especially anthocyanin biosynthesis, in plums. Further studies are needed to determine whether the identified candidate genes are related to anthocyanin biosynthesis in plum.

## Author contributions

This study was conceived by ZF and XY. The plant material preparation were carried out by ZF and SP. ZF, XY, CJ, and DZ analyzed the RNA-seq data. ZF, CJ, DZ, and SP performed the laboratory experiments and analyses. ZF drafted the manuscript. XY revised the manuscript. All authors read and approved the final manuscript.

### Conflict of interest statement

The authors declare that the research was conducted in the absence of any commercial or financial relationships that could be construed as a potential conflict of interest.

## Abbreviations

4CL: 4-coumaroyl:CoA-ligase
ANS/LDOX: anthocyanidin synthase/leucoanthocyanidin dioxygenase
bHLH: basic Helix-Loop-Helix
C4H: cinnamate-4-hydroxylase
CHI: chalcone isomerase
CHS: chalcone synthase
COG: Cluster of Orthologous Groups of proteins DFR, dihydroflavonol 4-reductase
DAF: days after flowering
F3H: flavanone 3-hydroxylase
F3′H: flavonoid 3′-hydroxylase
FPKM: Fragments Per Kilobase of transcript per Million mapped reads
GO: gene ontology
GST: glutathione S-transferase
KEGG: Kyoto Encyclopedia of Genes and Genomes
nr: NCBI nonredundant protein database
PAL: phenylalanine ammonialyase
qRT-PCR: real-time quantitative RT-PCR
UFGT: UDP-glucose: flavonoid 3-O-glucosyltransferase.
