# Supplementary material for: Identification of Candidate Anthocyanin-Related Genes by Transcriptomic Analysis of ‘Furongli’ Plum (Prunus salicina Lindl.) during Fruit Ripening Using RNA-Seq
Source: Front Plant Sci. 2016 Aug 31;7:1338. doi: 10.3389/fpls.2016.01338 (PMC5005409; doi:10.3389/fpls.2016.01338)
Supplement: Supplementary file 1 [file Table1.DOC]

**Table S1.** Primer sequences for qRT-PCR analysis.

| Genes | Unigene ID | Forward primer | Reverse primer | Annealing  temperature |
| --- | --- | --- | --- | --- |
| *PAL* | c38398.graph_c0 | GCAACCAACTCCATCAAGCA | ACCTTGACCCCAGAGTCATG | 55 |
| *C4H* | c23939.graph_c0 | GCTCAGCAGAAGGGAGAGAT | AAGCTTCTGGATGTCTGGCT | 55 |
| *4CL* | c30378.graph_c0 | TTTGAGATCGGGACACTGCT | TTCTAACCACTGTCCCGCAT | 55 |
| *CHS* | c37054.graph_c0 | CTTTGGGCATCTCGGATTGG | CCAGTCCATCTCCTGTGGTT | 55 |
| *CHI* | c28749.graph_c0 | TTGGCGTGTACTTGGAGGAT | GTATTGCTGGCCGGTTAGTG | 55 |
|  | c8193.graph_c0 | GGGTCATGGGATCACTGACA | GCACCCCATATTGAGACCCT | 56 |
| *F3H* | c23888.graph_c0 | TGTGGAGGCTTGTGAGGATT | ATCTTGCACAGCTTCTCCCT | 55 |
| *DFR* | c24831.graph_c0 | GGGCCGCTACATTTGTTCTT | GCTCTGCAGGTATCAACAGC | 56 |
| *F3’H* | c38186.graph_c0 | AGATGATGGTGTTGGCAGGA | GTCAGCATCCTCTTTGAGCG | 55 |
|  | c25083.graph_c0 | TGTTCTTGCTTAGCGGGGTA | CCCCTGCCACAAATTTCTCC | 55 |
| *LDOX* | c29583.graph_c0 | AAGAGTTGAGACCTTGGCCA | GCCTTCTTCAACTCCTCCCT | 55 |
| *UFGT* | c19095.graph_c0 | GAGTCCCTTGGCTTCCTCTT | AGTTGGCCCATTTGATGCAG | 55 |
| *GST* | c29416.graph_c0 | ACTTCAACTTCTGGTGCTGC | CCCTGGAAGATGGCTCAGAT | 55 |
| *MYB* | c39005.graph_c0 | CTGGCTTCTGGACGCTTAAC | GGTGCCACTTTCCTTCTCCT | 56 |
| *MYBD* | c28480.graph_c0 | GCACCAAGAAACAGCAACAGC | CACCGGATGAAGTGGAATCGG | 55 |
| *bHLH* | c36695.graph_c0 | CTCGCCAAAATCTCACGAGG | ACGTGCCTCAAGATCCTGAA | 55 |
|  | c33382.graph_c0 | ATCTGCAACTCCAAGTCCCGC | CAGAGCACCACTACGTCCGTG | 58 |
| *NAC* | c27539.graph_c0 | CCGATGGAGGATCAGAGGAC | ACTGGTCCTGCATCAAGACA | 55 |
|  | c19209.graph_c0 | ATCAGTCCTAGTGCCATGGG | TGAAGTTTGCAAGGCTGACC | 55 |
| *ACTIN* | c38189.graph_c0 | TCCAGCAGCTTCCATTCCAAT | TTGCCCTGGACTATGAGCAAG | 56 |
